# Analysis of Prevalence and Mortality Among Neonates and Children With Intestinal Atresia: A Multinational Study, 1974–2015

**DOI:** 10.1002/bdr2.70032

**Published:** 2026-04-20

**Authors:** Angie Carreño, Maria Paula Aguilera, Lina Ibañez, Karen Sarmiento, Juan A. Gili, Csaba Siffel, Wendy N. Nembhard, Jorieke E. H. Bergman, Eva Bermejo‐Sánchez, Giovanna Tagliabue, Saeed Dastgiri, Marcia L. Feldkamp, Stephanie Pocius, Miriam Gatt, Laura Martínez, María Aurora Canessa, Boris Groisman, Karin Källén, Danielle Landau, Nathalie Lelong, Margery Morgan, Jazmín Arteaga‐Vázquez, Michele Santoroi, Anke Rissmann, Antonin Sipek, Elena Szabova, Wladimir Wertelecki, Mark A. Canfield, Pierpaolo Mastroiacovo, Ignacio Zarante

**Affiliations:** ^1^ Birth Defect Surveillance Program, Faculty of Health Sciences Pontificia Universidad Javeriana Cali Colombia; ^2^ Human Genetics Institute, Faculty of Medicine Pontificia Universidad Javeriana Bogotá Colombia; ^3^ Faculty of Health Sciences Universidad Libre Sectional Cali Cali Colombia; ^4^ Department of Physiologic Science, Faculty of Medicine Pontificia Universidad Javeriana Bogotá Colombia; ^5^ Latin American Collaborative Study of Congenital Malformations (ECLAMC), Center for Medical Education and Clinical Research (CEMIC‐CONICET) Buenos Aires Argentina; ^6^ National University of Villa María Córdoba Argentina; ^7^ College of Allied Health Sciences, Augusta University Augusta Georgia USA; ^8^ Arkansas Center for Birth Defects Research and Prevention and Arkansas Reproductive Health Monitoring System, University of Arkansas for Medical Sciences, Department of Epidemiology Little Rock Arkansas USA; ^9^ Department of Genetics EUROCAT Northern Netherlands, University of Groningen, University Medical Center Groningen Groningen the Netherlands; ^10^ ECEMC (Spanish Collaborative Study of Congenital Malformations), UIAC (Research Center on Congenital Anomalies) Institute of Rare Diseases Research (IIER), Instituto de Salud Carlos III Madrid Spain; ^11^ Lombardy Congenital Anomalies Registry, Cancer Registry Unit, Fondazione IRCCS, National Cancer Institute Rome Italy; ^12^ Health Services Management Research Centre, Tabriz University of Medical Sciences Tabriz Iran; ^13^ Division of Medical Genetics, Department of Pediatrics University of Utah School of Medicine Salt Lake City Utah USA; ^14^ Utah Birth Defect Network, Office of Children With Special Health Care Needs, Division of Family Health, Utah Department of Health and Human Services Salt Lake City Utah USA; ^15^ Malta Congenital Anomalies Registry, Directorate for Health Information and Research La Valeta Malta; ^16^ Genetics Department University Hospital Dr. José E. González, Autonomous University of Nuevo León Nuevo León Mexico; ^17^ Regional Register Congenital Malformation Maule Health Service (RRMC‐SSM) Maule Chile; ^18^ National Network of Congenital Anomalies of Argentina (RENAC), National Center of Medical Genetics, National Administration of Laboratories and Health Institutes (ANLIS), National Ministry of Health and Social Development Buenos Aires Argentina; ^19^ National Board of Health and Welfare Stockholm Sweden; ^20^ Department of Neonatology Soroka Medical Center Beer‐Sheva Israel; ^21^ University of Paris, CRESS Obstetrical, Perinatal and Pediatric Epidemiology Research Team (EPOPé), INSERM, INRA Paris France; ^22^ CARIS, the Congenital Anomaly Register for Wales, Singleton Hospital Swansea Wales UK; ^23^ Department of Genetics, RYVEMCE National Institute of Medical Sciences and Nutrition Salvador Zubirán Mexico City Mexico; ^24^ Unit of Epidemiology of Rare Diseases and Congenital Anomalies Institute of Clinical Physiology, National Research Council Pisa Italy; ^25^ Malformation Monitoring Centre Saxony‐Anhalt, Medical Faculty Otto‐von‐Guericke University Magdeburg Germany; ^26^ Department of Medical Genetics Thomayer Hospital Prague Czech Republic; ^27^ Slovak Teratologic Information Centre (FPH), Slovak Medical University Bratislava Slovak Republic; ^28^ Omni‐Net for Children International Charitable Fund Rivne Ukraine; ^29^ Birth Defects Epidemiology and Surveillance Branch, Texas Department of State Health Services Austin Texas USA; ^30^ International Center on Birth Defects, International Clearinghouse for Birth Defects Surveillance and Research Rome Italy; ^31^ San Ignacio University Hospital Bogotá Colombia

**Keywords:** epidemiology, mortality, small intestinal atresia, surveillance programs, surveillance registries

## Abstract

**Introduction:**

Small intestinal atresia (SIA) consists of a congenital obstruction of the lumen of the duodenum, jejunum, or ileum with varying severity. The aim of the investigation was to analyze the prevalence and mortality of SIA, using data from the International Clearinghouse for Birth Defects Surveillance and Research (ICBDSR).

**Methods:**

Data on SIA cases were collected from 25 ICBDSR members' surveillance programs in 17 countries over 1974–2015. All pregnancy outcomes were included, but terminations of pregnancy were not available for 11 programs. Statistical analysis is descriptive, and the prevalence is established by the total of SIA cases divided by the total of births. The survival time was calculated, and mortality was analyzed individually using the Kaplan–Meier method for comparison.

**Results:**

The total prevalence of SIA was 2.1 per 10,000 births. Iran had the highest prevalence with 11.5 per 10,000 total births (95% CI: 9–14.1); on the other hand, the lowest prevalence of SIA was in Mexico‐Nuevo Leon with 0.5 per 10,000 births (95% CI: 0.3–0.8), and Cali‐Colombia had zero cases. In South America, a higher prevalence of SIA was estimated compared to what was reported in 2000. Most deaths occurred between Day 2 and 6, except in Bogotá‐Colombia, Spain, UK‐Wales, and Mexico, where the deaths occurred on Day 1. The mortality in the first year was 4.3%, but the specific causes of death were not determined in this study.

**Conclusion:**

The prevalence of SIA was about 2.1 per 10,000 births during a 41‐year period in 25 centers, with variations in prevalence according to geographical locations. Future research is suggested to analyze changes in trends and the impact of early diagnosis and treatment in mortality.

## 1 Introduction

Small intestinal atresia or severe stenosis (SIA) is a congenital obstruction of the duodenum, jejunum, or ileum, ranging from luminal narrowing to complete discontinuity of the intestine and mesentery (Rich et al. 2022). Duodenal atresia results from failed recanalization during early gestation, whereas jejunoileal atresia usually arises from late vascular disruption (Kulkarni 2010). Clinical manifestations depend on the level and severity of obstruction: duodenal atresia typically presents with bilious vomiting and early feeding intolerance, while jejunoileal forms manifest later.

SIA often co‐occurs with other anomalies, and the proportion of associated defects varies by anatomic site—more frequent in duodenal (18%–76%) than in jejunoileal atresia (13%–52%) (Hemming and Rankin 2007; Burjonrappa et al. 2011; Best et al. 2012; Takahashi et al. 2014; Miscia et al. 2018; Bethell et al. 2019). Small intestinal atresia may also be associated with other severe congenital conditions, such as biliary atresia, which significantly increases morbidity and mortality, particularly due to complications such as sepsis and liver failure (Jiang et al. 2022). Duodenal atresia is commonly associated with Down syndrome, while jejunoileal atresia correlates with gastroschisis (Kulkarni 2010; Burjonrappa et al. 2011; Gutiérrez‐Carrillo et al. 2013; Takahashi et al. 2014; Miscia et al. 2018; Bethell et al. 2019). Chromosomal abnormalities occur in up to 25% of all SIA and 55.0% of duodenal cases (Hemming and Rankin 2007; Best et al. 2012; Takahashi et al. 2014).

Reported prevalence of SIA ranges between 1 and 3 per 10,000 births, with similar rates in Europe and North America (Martínez‐Frías et al. 2000; Forrester and Merz 2004; Hemming and Rankin 2007; Best et al. 2012; Lupo et al. 2017). The NCARDRS (2019) and European Platform on Rare Disease Registration (EUROCAT) (2019) networks reported prevalences of 1.8 and 1.36 per 10,000 births for duodenal atresia, respectively. Also, in the UK, the total prevalence for SIA cases since 1991 to 2001 was 2.66 per 10,000 births (95% CI: 2.13–3.18) (Hemming and Rankin 2007).

In Latin America, the ECEMC and ECLAMC programs observed comparable rates of 1.3 per 10,000 live births (Martínez‐Frías et al. 2000). Duodenal atresia represents approximately 60% of SIA, with a jejunoileal to duodenal ratio near 2:1 (Rich et al. 2022).

Mortality remains heterogeneous across regions. In high‐income settings, survival exceeds 90%, as reported by EUROCAT (Glinianaia et al. 2022), while in low‐ and middle‐income countries mortality may surpass 40%, largely due to delayed diagnosis, limited perioperative care, and resource constraints (Gutiérrez‐Carrillo et al. 2013; Cairo et al. 2017).

Most existing data on SIA prevalence and survival derives from single or regional registries. Therefore, this study aimed to assess the prevalence and age‐specific mortality of SIA over an extended period data from multiple programs affiliated with the International Clearinghouse for Birth Defects Surveillance and Research (ICBDSR).

## 2 Methods

### 2.1 Study Design and Setting

This retrospective study used aggregated data provided by 25 congenital anomaly surveillance programs that are members of the International Clearinghouse for Birth Defects Surveillance and Research (ICBDSR), representing 17 countries across Asia, Europe, North America, and South America. Importantly, while the ICBDSR includes programs from 17 countries, several countries contribute to multiple regional or subnational registries, not single national systems (International Clearinghouse for Birth Defects Surveillance and Research (ICBDSR) n.d.).

For instance, Colombia contributed data from two regional hospital‐based programs (Bogotá y Cali), as well as the multi‐country ECLAMC network, which includes centers from Argentina, Bolivia, Brazil, Chile, Colombia, Costa Rica, Dominican Republic, Ecuador, Paraguay, Peru, Uruguay, and Venezuela (Estudio Colaborativo Latino Americano de Malformaciones Congénitas (ECLAMC) n.d.).

Mexico was represented by two regional programs (Nuevo León and RYVEMCE), Italy by two regional population‐based registries (Lombardy and Tuscany), Spain contributed two hospital‐based registries of the ECEMC network (with and without TOPFA data), while Sweden provided two time‐defined national registries (before and after legalization of TOPFA). United States included three population‐based state programs (Arkansas, Texas, and Utah), and United Kingdom contributed through the Wales CARIS regional registry.

In the regional category of Asia, the data included in the analysis originate exclusively from two participating programs: Iran and Israel. Although both countries are geographically located in Western Asia, they represent only a limited portion of the continent.

The remaining countries contributed national or single regional registries: Czech Republic, France (Paris), Germany (Saxony‐Anhalt), Malta, the Netherlands (Northern Netherlands), Slovak Republic, Ukraine (OMNI‐Net), Israel (Soroka Medical Center), and Iran (Tabriz TROCA).

Each program collects data on live births, stillbirths, and, when legally permitted, Elective Terminations of Pregnancy for Fetal Anomaly (ETOPFA), in accordance with their national definitions and protocols. Detailed characteristics of each program, including coverage type (national, regional, or hospital‐based), ascertainment period, and follow‐up methodology, are summarized in Tables 1 and 2.

**TABLE 1:** Characteristics of the surveillance programs.

Country—program	Period (years studied)	Type of program	Type of coverage	Stillbirth	ETOPFA	Period of diagnosis	Prenatal services included
*Asia*							
Iran—TROCA	2008–2012 (5)	Hospital‐based	Regional	20 weeks	Not reported	1 year	Yes
Israel—IBDSP/SMC	2000–2014 (15)	Hospital‐based	Regional^c^	Not included	Not reported	Hospital discharge	Yes
*Europe*							
Czech Republic—NR	1974–2014 (41)	Population‐based	National	22 weeks or ≥ 500 g	Allowed	15 years	Yes
France—Paris	1981–2014 (34)	Population‐based	Regional	22 weeks	Allowed	28 days	Yes
Germany—Saxony Anhalt	1980–2014 (35)	Population‐based	Regional	≥ 500 g	Allowed	1 year	Yes, since 1990
Italy—Lombardy	2003–2012 (10)	Population‐based	Regional	23 weeks	Allowed	6 years	Yes
Italy—Tuscany	1992–2014 (23)	Population‐based	Regional	20 weeks	Allowed	1 year	Yes
Malta—MCAR	1995–2013 (19)	Population‐based	National	22 weeks	Not allowed	1 year	Yes, gradually
Netherlands—North	1981–2014 (34)	Population‐based	Regional	24 weeks	Allowed	10 years	Yes, since 2007
Slovakia—STIC	2001–2014 (14)	Population‐based	National	≥ 500 g	Allowed	1 year	Yes
Spain—ECEMC‐TOPFA	1995–2013 (19)	Hospital‐based	Regional^b^	24 weeks or ≥ 500 g^d^	Allowed^e^	3 days	Yes
Spain—ECEMC‐No TOPFA	1980–2013 (34)	Hospital‐based	Regional^b^	24 weeks or ≥ 500 g	Not reported	3 days	Yes
Sweden—No TOPFA	1974–1998 (25)	Population‐based	National	Until 2006: 28 weeks, 2007 and after: 22 weeks	Not allowed	Before 1987: 28 days, After 1987: 1 year	Yes, since early 1980s
Sweden—TOPFA	1999–2014 (16)	Population‐based	National	Until 2006: 28 weeks, 2007 and after: 22 weeks	Allowed	1 year	Yes, since early 1980s
Ukraine—OMNI‐net	2000–2013 (14)	Population‐based	Regional	Until 2006: 28 weeks or ≥ 1000 g, 2006 and after: 22 weeks	Allowed	1 year	Yes
UK—Wales CARIS	1998–2014 (17)	Population‐based	Regional	24 weeks	Allowed	18 years	Yes, since 2003
*North America*							
Mexico—Nuevo Leon	2011–2015 (5)	Population‐based	Regional	Not included	Not allowed	6 days	Yes, only US
Mexico—RYVEMCE	1978–2013 (36)	Hospital‐based	Regional	≥ 20 weeks or ≥ 500 g	Not allowed	3 days	No
USA—Arkansas	1993–2012 (20)	Population‐based	Statewide	20 weeks	Allowed	2 years	Yes
USA—Texas	1996–2012 (17)	Population‐based	Statewide	20 weeks	Allowed	1 year	Yes
USA—Utah	1994–2012 (19)	Population‐based	Statewide	20 weeks	Allowed	2 years	Yes
*South America*							
Argentina—RENAC	2009–2014 (6)	Hospital‐based	National	≥ 500 g	Not allowed	Hospital discharge	Yes, no official program
Colombia—Bogotá	2000–2014 (15)	Hospital‐based	Regional	≥ 500 g	Not reported	Hospital discharge	Yes
Colombia—Cali	2011–2014 (4)	Hospital‐based	Regional	≥ 500 g	Not reported	Hospital discharge	Yes
South America—ECLAMC	1995–2014 (20)	Hospital‐based	Regional^a^	≥ 500 g	Not allowed	Hospital discharge	Yes

^a^ Several regions in South America.

^b^ Several regions in Spain currently cover around 18% of total births.

^c^ Referral area of one hospital.

^d^ Stillbirth definition applies if gestational age of death is not determined (since 1980).

^e^ Data only available from 1995 onwards.

**TABLE 2:** Characteristics of the follow‐up of the surveillance program.

Country—program	Follow‐up until discharge after death	Follow‐up by clinician or program staff	Linkage to death certificate	Maximum follow‐up
*Asia*				
Iran—TROCA	Yes	Yes^c^	No	1–6 days
Israel—IBDSP/SMC	Yes	No	Yes, 2000–2014	1–4 years
*Europe*				
Czech Republic—NR	Yes	Yes	Yes, 1974–2014	≥ 5 years
France—Paris	Yes	Yes	Yes, 1981–2014	28 days
Germany—Saxony Anhalt	Yes	No^b^	Yes, 1980–2014	≥ 5 years
Italy—Lombardy	Yes	Yes	No	1–6 days
Italy—Tuscany	Yes	Yes	Yes, 1992–2015	28 days– 11 months
Malta—MCAR	Yes^a^	Yes	Yes, 1995–2013^d^	1–6 days
Netherlands—NNL	Yes	Yes	Yes, 1981–2014	≥ 5 years
Slovak Republic—SRC	Yes	Yes	Yes, 2001–2014	28 days–11 months
Spain—ECEMC (No TOPFA)	Yes	Yes	No	1 days
Spain—Catalunya (TOPFA)	Yes	Yes	No	1–6 days
Sweden (No TOPFA)	Yes	Yes	Yes, until 1998	1 year
Sweden (TOPFA)	Yes	Yes	Yes, 1999–2014	1 year
UK—Wales (CARIS)	Yes	Yes	Yes, 2000–2013	≥ 5 years
Ukraine—OMNI‐net	Yes	Yes	Yes, 1998–2014	≥ 5 years
*North America*				
Mexico—Nuevo Leon	Yes	No	No	1–6 days
Mexico—RYVEMCE	Yes	No	No	1–6 days
USA—Arkansas	Yes	No	Yes, 1993–2015	≥ 5 years
USA—Texas	Yes	No	Yes, 1996–2013	≥ 5 years
USA—Utah	Yes	No	Yes, until age 2	≥ 5 years
*South America*				
Argentina—RENAC	Yes	Yes	No	1–6 days
Colombia—Bogotá	Yes	Yes	No	1 day
Colombia—Cali	Yes	Yes	No	No mortality reported (LB)
South America—ECLAMC	Yes	Yes	No	28 days–11 months

^a^ Babies are followed up until discharge and their hospital files are reviewed at 1 year of age, linkage with mortality data continues indefinitely.

^b^ Until 18 years.

^c^ Children in university hospital(s).

^d^ Continuous linkage with mortality register, for this study, data have linkage up to 2015.

The data provided were from the year the programs were initiated until 2014 or earlier, depending on the program (see Table 1). It included total births per year and cases of SIA categorized by pregnancy outcome (LB, stillbirths, or ETOPFA) and clinical presentation (Isolated, multiple congenital anomalies [MCA] or syndromic). MCA was defined as two or more major congenital anomalies in different organ systems, and syndromic was defined as having genetic or chromosomal abnormalities. Data on mortality among LB were provided by age at death: Day 1, 1 week, 1 month, 1 year, and 5 years of age. Mortality data were obtained through different follow‐up methods established by each program (see Table 2).

### 2.2 Case Definition

For this study, different surveillance programs from Asia, Europe, North and South America provided data on SIA using the International Classification of Disease (ICD)‐10 code Q41 and ICD‐9 code 751.1.

### 2.3 Statistical Analysis

Descriptive statistics were calculated for the main variables of the study, including both epidemiological and clinical indicators. These variables comprised:

1. Type of atresia (duodenal, jejunal, or ileal);
2. Geographical origin (country and program/region, as listed in Tables 1 and 2);
3. Sex of the newborn;
4. Presence and type of associated anomalies, classified as chromosomal (e.g., trisomy 21), syndromic, or structural nonsyndromic.
5. Pregnancy outcome, including live births, stillbirths, and Elective Terminations of Pregnancy for Fetal Anomaly (ETOPFA) when available;
6. Period of diagnosis or birth cohort; and
7. Mortality by age group, assessed through survival time from birth up to 1 year of age.

For each variable, absolute and relative frequencies were calculated, and prevalence rates were expressed per 10,000 total births with 95% confidence intervals. Mortality was analyzed using the Kaplan–Meier method to estimate survival functions, and comparisons across programs and regions were descriptive, without inferential testing due to differences in data availability and ascertainment procedures among registries.

Descriptive statistics were calculated for the main variables of the study. The data were evaluated and presented in frequency tables with proportions. The total prevalence was calculated as the total number of cases of SIA divided by the total number of births (LB + stillbirths + ETOPFA, when data are available). Prevalence among LB, stillbirths, and ETOPFA in each program was also calculated.

We determined the proportion of infants with SIA surviving to 1 month (including programs reporting survival to hospital discharge, 1 week or 28 days), 1 year and 5 years after birth, based on the number of LB infants with SIA. We calculated overall survival at 1 month, 1 year, and 5 years for all contributing programs, for cases of isolated SIA, and for SIA occurring with an additional major anomaly or with chromosomal/genetic diagnoses. To compare overall 1‐month to 1‐year survival, we restricted the analysis to programs with survival data up to 1 year. Similarly, when comparing 1 month to 5‐year survival, we limited analyses to programs and birth cohorts with survival data up to 5 years. For this comparison, a restricted cohort for each program was defined by years of birth to ensure a complete 5‐year follow‐up.

The mortality was evaluated for each program and each clinical presentation individually. Eighteen programs provided information for each group, one program provided information for the syndromic group only, and six programs could not classify the cases. The Kaplan–Meier method was used to estimate the survival curves and compare the cumulative probability of no event of death in each of the programs.

### 2.4 Ethics

Each program had locally approved ethics procedures, and because this study was conducted using aggregated data, no additional ethics committee approval was required.

### 2.5 Patient Consent Statement

In the Latin American countries associated with ECLAMC, written informed consent was obtained from all study participants and, in the case of minors, from their legal guardians. In some of these countries, patients or their legal guardians remain in the program unless they explicitly request to be removed. Since 1992, the EUROCAT NNL program has required parental consent for data collection, whereas before that year, it was not necessary under local ethical regulations. Since the implementation of this policy, most parents have provided consent for participation. In contrast, in many other ICBDSR member programs, cases remain under passive surveillance unless parents explicitly request removal from the registry, in accordance with national data protection policies.

## 3 Results

Of the 25 ICBDSR member programs provided data (Table 1), 9 were hospital‐based and 16 were population‐based (PB). Out of all programs, 24% (*n* = 6) had national coverage, 64% (*n* = 16) had regional, and 12% (*n* = 3) had statewide coverage. The ascertainment period and stillbirth definition varied among programs. ETOPFA is not allowed in six programs (Argentina‐RENAC, Malta‐MCAR, Mexico‐Nuevo Leon, Mexico RYVEMCE, South America ECLAMC, and Sweden before 1999), and five programs did not report it (Colombia—Bogota, Colombia—Cali, Iran—TROCA, Israel—IBDSP/SMC, and Spain ECEMC—[No TOPFA]). The regions that mostly allow ETOPFA are Europe and North America except for the programs Malta‐MCAR, Spain ECEMC [No TOPFA], Sweden‐No TOPFA, Mexico‐Nuevo Leon, and Mexico RYVEMCE. Twenty programs provided follow‐up until discharge after birth, 10 programs have follow‐up by clinician or program staff, and 11 programs have a linkage to death certificate. The maximum follow‐up time is heterogeneous, ranging from 1 day to 5 years of age (Table 2).

All programs combined, from 1974 to 2015, reported 6305 SIA cases among 30,563,026 births, resulting in a total SIA prevalence of 2.1 per 10,000 births (95% CI: 2.0–2.2). The highest prevalence of SIA was found in Iran with 11.5 per 10,000 total births (95% CI: 9–14.1) followed by the Slovak Republic (10.3 per 10,000 births, 95% CI: 8.8–12.0). The lowest prevalence of SIA was in Mexico‐Nuevo Leon with 0.5 per 10,000 births (95% CI: 0.3–0.8) and Cali, Colombia where there was no SIA case during the surveillance period (Table 3).

**TABLE 3:** Total number of births, total number of small intestinal atresia cases and prevalence per 10,000 births, proportion of livebirth among total cases of small intestinal atresia, proportion of stillbirths among total cases of small intestinal atresia, and proportion of ETOPFA among total cases of small intestinal atresia from programs contributing to the International Clearinghouse for Birth Defects Surveillance and Research.

Country—program	Type of program	Surveillance periods	Total births	Total cases of small intestinal atresia (SIA)	Total prevalence per 10,000 total birth (95% CI)	Livebirth% (95% CI)	Stillbirth% (95% CI)	ETOPFA% (95% CI)
*Asia*								
Iran—TROCA	H	2008–2012	78,446	90	11.5 (9.2–14.1)	98.9 (93.9–99.9)	1.1 (0.0–6.0)	—
Israel—SMC^a^	H	2000–2014	200,660	38	1.9 (1.3–2.6)	100.0	0.0	—
*Europe*								
Czech Republic	P	1974–2014	5,146,979	592	1.2 (1.1–1.3)	95.9 (94.0–97.4)	0.5 (0.1–1.5)	3.5 (2.2–5.4)
France—Paris	P	1981–2014	875,241	250	2.9 (2.5–3.2)	82.0 (76.7–86.6)	4.4 (2.2–7.7)	13.6 (9.6–18.5)
Germany	P	1980–2014	526,289	85	1.6 (1.2–2.0)	92.9 (85.3–97.4)	4.7 (1.3–11.6)	2.4 (0.3–8.2)
Italy—Lombardy	P	2003–2012	133,182	32	2.4 (1.6–3.4)	100.0	0.0	—
Italy—Tuscany	P	1992–2014	636,562	102	1.6 (1.3–2.0)	94.1 (87.6–97.8)	4.9 (1.6–11.1)	1.0 (0.0–5.3)
Malta—MCAR^a^	P	1995–2013	79,948	14	1.8 (1.0–3.0)	85.7 (57.2–98.2)	14.3 (1.9–42.8)	—
Netherlands—Northen	P	1981–2014	562,462	126	2.2 (1.9–2.7)	88.9 (82.1–93.8)	4.8 (1.8–10.8)	6.4 (2.8–12.1)
Slovak Republic	P	2001–2014	165,900	170	10.3 (8.8–12.0)	97.1 (93.3–99.0)	2.4 (0.7–5.9)	0.6 (0.0–3.2)
Spain—ECEMC^a^	H	1980–2013	2,891,337	306	1.1 (1.0–1.2)	97.4 (94.9–98.9)	2.6 (1.1–5.1)	—
Spain—TOP	H	1995–2013	373,698	47	1.3 (1.0–1.7)	78.7 (64.3–89.3)	2.1 (0.1–11.3)	19.1 (9.2–33.3)
Sweden	P	1974–2014	4,195,523	1239	3.0 (2.8–3.1)	97.7 (96.7–98.4)	1.1 (0.6–1.9)	1.2 (0.7–2.0)
Ukraine—OMIN‐Net	P	2000–2013	404,172	125	3.1 (2.6–3.7)	92.8 (86.8–96.7)	4.0 (1.3–9.1)	3.2 (0.9–8.0)
UK—Wales	P	1998–2014	596,341	179	3.0 (2.6–3.5)	92.7 (88.0–96.1)	1.7 (0.4–4.8)	5.6 (2.7–10.0)
*North America*								
Mexico—Nuevo Leon^a^	P	2011–2015	442,674	22	0.5 (0.3–0.8)	100.0	0.0	—
Mexico—RYVEMCE^a^	H	1978–2013	1,198,579	151	1.3 (1.7–1.9)	99.3 (96.4–99.9)	0.7 (0.0–3.6)	—
USA—Arkansas	P	1993–2012	760,777	274	3.6 (3.2–4.1)	94.9 (91.6–97.2)	5.1 (2.8–8.4)	—
USA—Texas	P	1996–2012	5,980,798	1078	1.8 (1.7–1.9)	99.1 (98.3–99.6)	0.7 (0.3–1.5)	0.2 (0.0–0.7)
USA—Utah	P	1994–2012	928,107	104	1.1 (0.9–1.4)	95.2 (89.1–98.4)	1.9 (0.2–6.8)	2.9 (0.6–8.2)
*South America*								
Argentina—RENAC^a^	H	2009–2014	1,023,108	315	3.1 (2.8–3.4)	99.0 (97.2–99.8)	1.0 (0.2–2.8)	—
Colombia—Bogotá^a^	H	2000–2014	407,394	43	1.1 (0.8–1.4)	95.3 (84.2–99.4)	4.7 (0.6–15.8)	—
Colombia—Cali^a^	H	2011–2014	27,294	0	0.0 (0.4–1.4)	0.0	0.0	—
South America—ECLAMC	H	1995–2014	2,927,555	923	3.2 (3.0–3.4)	97.1 (95.8–98.1)	2.9 (1.9–4.2)	—
Total			31,903,825	6759	2.1 (2.1–2.2)	96.3 (95.8–96.7)	2.0 (1.7–2.3)	1.7 (1.4–2.0)

Abbreviations: ECEMC, Registry of the Spanish Collaborative Study of Congenital Malformations; ECLAMC, Latin American Collaborative Study of Congenital Malformations; H, Hospital‐based programme; MCAR, Malta Congenital Anomalies Registry; OMNI‐Net, Ukraine Birth Defects Prevention Program; P, Population‐based programme; RENAC, National Network of Congenital Anomalies of Argentina; RYVEMCE, Mexican Registry and Epidemiological Surveillance of External Congenital Malformations; SB, Stillbirth; SMC, Soroka Medical Center; TROCA, Tabriz Registry of Congenital Anomalies; UK, United Kingdom; USA, United States of America.

^a^ TOPFA not allowed/not registered.

Hospital‐based programs had an average SIA prevalence of 2.10 per 10,000 births (95% CI: 2.00–2.19), very similar to population‐based programs (2.13 per 10,000 births; 95% CI: 2.07–2.19). When we compare prevalence by region, the average SIA prevalence of South America programs was 2.92 per 10,000 births (95% CI: 2.76–3.09), while in Europe the prevalence was 1.97 per 10,000 births (95% CI: 1.90–2.04), in North America 1.96 per 10,000 births (95% CI: 1.87–2.04), and in Asia programs (Iran and Israel) 4.59 per 10,000 births (95% CI: 3.38–5.45) (Table 3).

The overall percentage of LB infants with SIA was 96.3% (95% CI: 95.8%–96.7%), the percentage of stillbirth was 2.0% (95% CI: 1.7%–2.4%), and the percentage of ETOPFA among SIA cases was 1.7% (95% CI: 1.4%–2.0%), with the highest percentage of ETOPFA in Paris, France 13.6% (95% CI: 9.6%–18.5%) (Table 3).

Most deaths occurring between Day 2 and Day 6 (3.0%, 95% CI: 2.6%–3.4%), except in Bogotá‐Colombia, Spain (ECEMC), UK‐Wales and Mexico (RYVEMCE) where most deaths occurred on the first day. Between Day 28 and Month 11, the mortality was 4.3% (95% CI: 3.8%–4.8%), with high mortality (50%) observed in Mexico‐Nuevo León (Tables 4 and 5).

**TABLE 4:** Mortality in small intestinal atresia‐affected births for surveillance period 1974–2014 from programs contributing to the International Clearinghouse for Birth Defects Surveillance and Research.

Country—program	Surveillance period	Livebirths with small intestinal atresia	Age at death					
			Day 1	Day 2–Day 6	Day 7–Day 27	Day 28–Month 11	Year 1–4	Year 5 and above
		*N*	%	%	%	%	%	%
*Asia*								
Iran—TROCA	2008–2012	89	0.0	0.0	—	—	—	—
Israel—SMC^a^	2000–2014	38	0.0	0.0	0.0	10.5	0.0	0.0
*Europe*								
Czech Republic	1974–2014	568	0.2	0.9	2.8	5.6	0.7	0.90
France—Paris	1981–2014	205	2.0	3.4	2.4	—	—	—
Germany—Saxony Anhalt	1980–2014	79	0.0	2.5	2.5	5.1	0.0	0.0
Italy—Lombardy	2003–2012	32	0.0	3.1	0.0	3.1	6.3	—
Italy—Tuscany	1992–2014	96	1.0	1.0	3.1	—	—	—
Malta—MCAR^a^	1995–2013	12	0.0	8.3	8.3	0.0	0.0	0.0
Netherlands—Northern	1981–2014	112	0.9	5.4	2.7	4.5	0.0	0.9
Slovak Republic	2001–2014	165	0.0	5.5	4.8	—	—	—
Spain—ECEMC^a^	1980–2013	298	2.7	2.3	—	—	—	—
Spain—TOP	1995–2013	37	0.0	0.0	—	—	—	—
Sweden	1974–2014	1210	1.5	2.7	2.2	6.4	1.2	0.9
Ukraine—OMNI‐Net	2000–2013	116	3.4	12.9	9.5	18.1	0.9	—
UK—Wales	1998–2014	166	2.4	1.8	1.2	4.2	1.2	0.6
*North America*								
Mexico—Nuevo Leon^a^	2011–2015	22	4.5	18.2	4.5	50.0	13.6	4.5
Mexico—RYVEMCE^a^	1978–2013	150	5.3	4.7	—	—	—	—
USA—Arkansas	1993–2012	260	0.4	1.5	2.3	4.6	1.2	1.5
USA—Texas	1996–2012	1068	0.3	0.7	1.2	5.1	1.5	0.5
USA—Utah	1994–2012	99	1.0	1.0	2.00	2.0	1.0	0.0
*South America*								
Argentina—RENAC^a^	2009–2014	312	0.0	8.3	—	—	—	—
Colombia—Bogotá^a^	2000–2014	41	2.4	0.0	—	—	—	—
Colombia—Cali^a^	2011–2014	0	0.0	0.0	—	—	—	—
South America—ECLAMC^a^	1995–2014	896	2.3	5.2	4.9	2.8	0.0	0.00
Total		6508	1.3	3.0	2.3	4.3	0.8	0.6

Abbreviations: ECEMC, Registry of the Spanish Collaborative Study of Congenital Malformations; ECLAMC, Latin American Collaborative Study of Congenital Malformations; MCAR, Malta Congenital Anomalies Registry; OMNI‐Net, Ukraine Birth Defects Prevention Program; RENAC, National Network of Congenital Anomalies of Argentina; RYVEMCE, Mexican Registry and Epidemiological Surveillance of External Congenital Malformations; SB, Stillbirth; SMC, Soroka Medical Center; TROCA, Tabriz Registry of Congenital Anomalies; UK, United Kingdom; USA, United States of America.

^a^ TOPFA not allowed/not registered.

**TABLE 5:** Type of birth and first‐week mortality among live births affected with small intestinal atresia according to clinical presentation from programs contributing to the International Clearinghouse for Birth Defects Surveillance and Research, 1974–2014.

Country—program	Isolated small intestinal atresia							Multiple/syndromic small intestinal atresia						
	Total cases		Type of birth			Mortality in LB		Total cases		Type of birth			Mortality in LB	
			ETOPFA	SB	LB	Day 1	Day 2–6			ETOPFA	SB	LB	Day 1	Day 2–6
	*N*	%	%	%	%	%	%	*N*	%	%	%	%	%	%
*Asia*														
Israel—SMC	37	97.4	00.0	00.0	100.0	00.0	00.0	1	2.6	—^a^	00.0	100.0	00.0	00.0
*Europe*														
France—Paris	155	62.0	0.0	3.2	96.8	0.7	2.0	95	38.0	35.8	6.3	5.9	5.5	7.3
Germany—Saxony Anhalt	63	74.1	0.0	3.2	96.8	0.0	1.6	22	25.9	9.1	9.1	81.8	0.0	5.6
Italy—Lombardy	13	40.7	0.0	0.0	100.0	0.0	0.0	19	59.4	0.0	0.0	100.0	00.0	4.2
Malta—MCAR	9	64.3	0.0	0.0	100.0	0.0	11.1	5	35.7	—^a^	40.0	60.0	0.0	0.0
Netherlands—Northern	78	61.9	00.0^a^	3.8	96.2	0.0	1.3	48	38.1	16.7	6.3	77.1	2.7	13.5
Slovak Republic	109	64.1	0.0	2.8	97.3	0.0	0.94%	61	35.9	1.6	0.0	98.4	00.0	15.0
Spain—ECEMC	137	44.8	00.0	0.7	99.3	0.7	00.0	169	55.2	0.0	4.1	95.9	4.3	4.3
Spain—TOP	19	40.4	5.3	00.0	94.7	00.0	00.0	28	59.6	28.7	3.6	67.9	00.0	00.0
Sweden	756	61.0	00.0	0.9	99.1	0.4	1.6	483	39.0	3.1	1.5	95.5	3.3	4.6
Ukraine—OMNI‐Nete	80	64.0	1.3	1.3	97.5	3.9	10.3	45	36.0	6.7	8.9	84.4	2.6	18.4
UK—Wales	103	57.5	00.0	1.0	99.0	1.0	1.0	76	42.5	11.8	1.3	86.8	3.0	3.0
*North America*														
Mexico—RYVEMCE	88	58.3	00.0	00.0	100.0	2.3	3.4	63	41.7	—^a^	1.6	98.4	9.7	6.5
USA—Utah	87	83.7	2.3	2.3	95.4	1.2	1.2	17	16.4	5.9	00.0	94.1	00.0	00.0
*South America*														
Argentina—RENAC	162	51.4	0.00	1.2	98.8	—	5.6	153	48.6	—^a^	0.6	99.4	—	11.2
Colombia—Bogotá	35	81.4	0.00	4.7	95.3	2.86	—	8	18.6	—^a^	25.0	75.0	0.0	—
South America—ECLAMC	463	50.2	0.0	2.2	97.8	0.2	3.8	460	49.8	—^a^	3.7	96.3	4.5	6.7
Total	2391	57.1	0.2	1.5	98.4	0.6	2.5	1795	42.9	4.6	3.1	92.6	3.3	6.6

Abbreviations: ECEMC, Registry of the Spanish Collaborative Study of Congenital Malformations; ECLAMC, Latin American Collaborative Study of Congenital Malformations; MCAR, Malta Congenital Anomalies Registry; OMNI‐Net, Ukraine Birth Defects Prevention Program; RENAC, National Network of Congenital Anomalies of Argentina; RYVEMCE, Mexican Registry and Epidemiological Surveillance of External Congenital Malformations; SB, Stillbirth; SMC, Soroka Medical Center; TROCA, Tabriz Registry of Congenital Anomalies; UK, United Kingdom; USA, United States of America.

^a^ TOPFA not allowed/not registered.

Among the cases with MCA/syndromic SIA 4.6% were ETOPFA, and 3.1% were stillbirths; in comparison with isolated cases 0.2% and 1.5%, respectively. MCA/syndromic SIA had higher mortality at Day 1 and 2–6 days (3.3%; 6.6%) compared with isolated SIA (0.6%; 2.5%) (Table 5).

**FIGURE 1:**
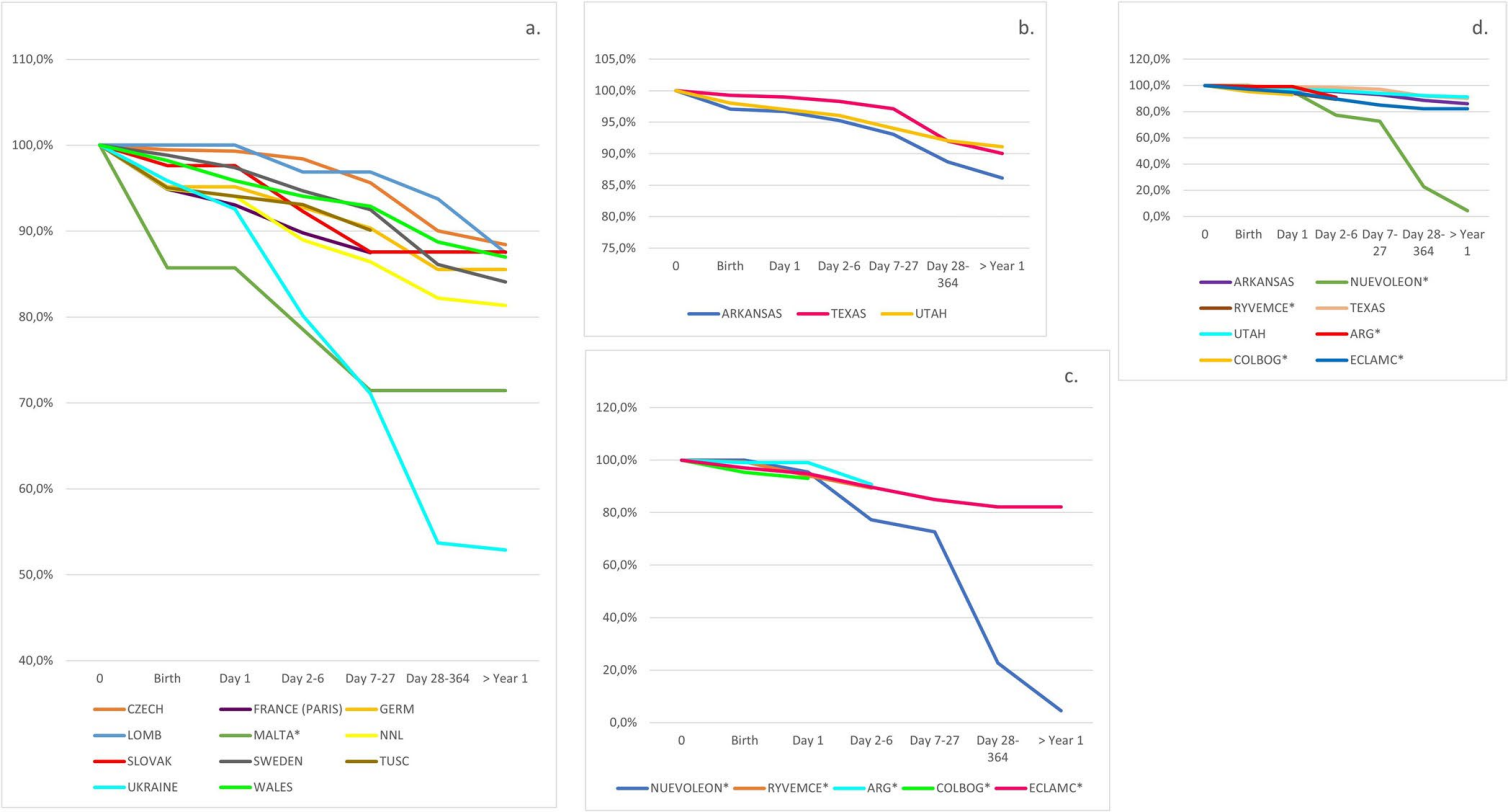
Survival in live births (LB) with small intestinal atresia for surveillance period 1974–2014, (a) from European programs; (b) from North American programs; (c) from Mexico and South America programs; (d) from North America (NA), South America and Europe programs contributing to the International Clearinghouse for Birth Defects Surveillance and Research.

Figure 1 presents the survival curves from 1974 to 2014 for total cases and by programs grouped by geographical region. European and North America's programs had the highest survival probability and South America programs had the lowest.

## 4 Discussion

This is one of the largest studies to assess the prevalence and mortality of SIA, using data from 25 programs of the ICBDSR over a 41‐year period. During this period the prevalence of SIA was 2.1 per 10,000 births. This rate falls within the range reported by other population‐based studies (Oddsberg et al. 2012); Takahashi et al. (2014) reported a prevalence of 2.23 (95% CI: 1.78–2.77) per 10,000 LB for SIA. However, Best et al. (2012) reported a prevalence of 1.6 per 10,000 births (95% CI: 1.5–1.7) for SIA. In our study, SIA prevalence varied between countries, from 0.5 per 10,000 births in Nuevo León, Mexico, to 11.5 per 10,000 births in Iran and 10.3 per 10,000 births in the Slovak Republic. The SIA prevalence in hospital‐based and population‐based programs was comparable (2.10 vs. 2.13 per 10,000 births, respectively).

To our knowledge, this is the first time a higher prevalence for SIA is reported in Iran. Farrokhkhani et al. (2023) evaluated during 2018–2022 in Northern Iran 169 patients with neonatal intestinal obstruction, where 16 had duodenal atresia (9.5%), 13 had jejunal atresia (7.7%), and 8 had ileal atresia (4.7%). The study conducted by Khaleghnejad‐Tabari et al. (2024) in Iran reported a prevalence of congenital digestive tract anomalies of 13.1 per 10,000 births (95% CI: 10.7–15.4), similar to the findings in our study. Data were collected from six main regions of Iran, where significant differences in the overall prevalence of congenital anomalies were observed. The highest rates were reported in the southern region (376.0 per 10,000 births; 95% CI: 350.9–402.4) and the northwestern region (356.4 per 10,000 births; 95% CI: 325.3–389.7), while the lowest prevalence was recorded in the southwestern region (94.3 per 10,000 births; 95% CI: 83.3–106.4). According to the authors, these variations may be associated with genetic, environmental, and socioeconomic factors, including a higher rate of consanguinity in some regions, differences in maternal exposure to teratogenic agents, and disparities in access to prenatal screening programs and advanced neonatal care.

Despite the lack of information in Asia, a study in India between 2010 and 2020 reported a frequency of 2.8% of 3672 cases of birth abnormalities have SIA and obstruction, this increase in frequency can be explained by the fact that atresia and intestinal obstruction were referenced together (Nanjunda et al. 2022). China has reported a prevalence of congenital intestinal obstruction of approximately 1:2000 live births, where duodenal atresia and stenosis are frequent causes of intestinal obstruction with a prevalence of 1 per 5000–10,000 live births, while jejunoileal atresia or stenosis is one of the leading causes of neonatal intestinal obstruction, with a prevalence of 6, 7 per 10,000 LB (“1:1500 LB”) to 30, 3 per 10,000 to (“1:330LB”) (Han et al. 2022). A case report from the Tawam Hospital (Al Ain, Abu Dhabi, United Arab Emirates) reported that congenital duodenal obstruction is an uncommon condition, with an estimated prevalence about 1 in 10,000–15,000 LB (Nawaz et al. 2005).

On the other hand, our study reported a higher prevalence for SIA in South America (3.2 per 10,000 births), compared with the data previously reported in 2000 covering the period 1967–1996 (1.29 per 10,000 births) (Martínez‐Frías et al. 2000). This increase may be due to a better understanding of the disease, improved diagnostic tools, and expanded prenatal and neonatal registries in the region, as well as better training of healthcare personnel, leading to earlier and more accurate diagnosis (Chirdan et al. 2004). However, this difference may also reflect changes in case ascertainment over time, which is a common challenge in birth defects surveillance and may influence prevalence estimates.

Differences in data collection and healthcare access across regions could influence reported prevalence and outcomes. South American programs showed a higher average prevalence of SIA compared to Europe and North America, possibly reflecting under‐ascertainment in earlier reports or differences in risk factors such as maternal health and nutrition. The Asian programs (here represented by Iran) showed the highest prevalence in this study, which may be related to genetic or environmental factors requiring further research.

Overall, the mortality of children born with SIA was 4.3% within the first year of life (suggesting a little over 95% survival rate). This is somewhat better than the survival rate for neonates in north‐west India reported by Gupta et al. in 2015 which is 63% (Gupta et al. 2016). Over the past three decades, the mortality rates of duodenal atresia have significantly dropped (averaging 2%–5%) and reporting the long‐term survival of most infants (more than 80%) with duodenal atresia is the norm (Sigmon et al. 2023). Moreover, the long‐term survival rate for children with SIA is from 60% to 100%, with colonic atresia having the best prognosis (Shaoying et al. 2008), which is similar to the findings of Glinianaia et al. (2022), in the study that was part of the EUROlinkCAT project, where the estimated 10‐year survival rate for children with duodenal atresia or stenosis or with atresia or stenosis of other parts of the small intestine was 93.6% and 94.1%, respectively (Glinianaia et al. 2022).

Information on the cause of death was not collected in our study. However, evidence suggests that mortality in these cases is primarily influenced by associated congenital abnormalities, such as complex cardiac defects or underlying genetic disorders (Sigmon et al. 2023; Garne et al. 2002). Nevertheless, among noncongenital causes of mortality, postoperative complications such as aspiration, anastomotic leak, and sepsis have been reported as significant contributors (Cairo et al. 2017). Additionally, studies indicate that SIA is more common in preterm infants and those with low birth weight (Gutiérrez‐Carrillo et al. 2013; Prasad and Bajpai 2000).

Other factors associated with higher mortality are the lack of resources or access to medical facilities, mostly in low‐income countries, below standard intraoperative and perioperative care, and lack of antenatal diagnosis (Chirdan et al. 2004; Global PaedSurg Research Collaboration 2021). A higher proportion of patients did not receive surgical intervention in low‐income countries compared with middle‐ and high‐income countries, and there is greater mortality from sepsis (Jiang et al. 2022) and respiratory failure in low‐income countries than in high‐income countries (Global PaedSurg Research Collaboration 2021). Therefore, this information cannot be reported or confirmed due to the nature of our study.

Overall survival of infants with SIA has improved over time, but disparities remain. Most infant deaths in this study occurred within the first week of life, often on the first day, highlighting the critical importance of neonatal surgical and intensive care. By 1 year of age, 4.3% of infants with SIA had died. Programs with limited resources (e.g., Mexico‐Nuevo León) showed significantly higher mortality (up to 50%), indicating that access to timely surgical intervention and postoperative care is a major determinant of survival.

In recent years, advances in perinatal medicine have improved the prenatal detection of SIA, enabling more effective prenatal counseling, the option of elective termination of pregnancy for fetal anomaly (ETOPFA), and the planning of delivery in specialized surgical centers (Bethell et al. 2019). In our study, 1.7% of cases were recorded as ETOPFA, 96.3% as live births (LB), and 2.0% as stillbirths. Among the cases undergoing ETOPFA, 4.6% corresponded to multiple congenital anomalies (MCA), 3.1% to cases with an identified syndrome, and the remaining cases to isolated forms of SIA. These findings highlight the influence of additional congenital anomalies on the decision to terminate pregnancy. In a study between 2008 and 2017 in the USA, there were no fetal or neonatal demises within the cases of isolated duodenal atresia, and only 1 of 7 isolated cases (14%) delivered at less than 32 weeks gestation (Bishop et al. 2020). According to this study, fetuses with isolated duodenal atresia on prenatal ultrasound generally have more favorable outcomes than the cases with additional anomalies.

About one third of the patients with syndromic SIA have trisomy 21 (Bethell et al. 2019), and those with a normal karyotype have a greater risk of having additional structural anomalies (Hemming and Rankin 2007; Best et al. 2012). According to previous studies, approximately 30%–40% of duodenal atresia cases are associated with trisomy 21. In these cases, duodenal atresia has been identified as a significant risk factor for perinatal mortality, contributing to a higher incidence of fetal and neonatal demise in this population (Adams and Stanton 2014; Bishop et al. 2020; Galani et al. 2021). The prevalence of duodenal atresia in Down syndrome is much higher than in the general population, with 3% prevalence of congenital duodenal atresia among these (Sigmon et al. 2023). Around 10%–25% of cases with duodenal atresia have structural cardiac and/or renal defects (Adams and Stanton 2014; Galani et al. 2021).

One of the strengths of this study is that we collected data with a standardized method to identify cases in all programs and cross‐checked from multiple reporting sources to ensure high case ascertainment, and some of the information provided was based on data derived from high‐quality, population‐based programs. Limitations include methodological variations between programs and limited access to clinical data such as risk factors, treatment, and cause of death, which could have enriched the study findings.

It's important to note that the causes of death were not detailed in our data, so we could not distinguish whether mortality was directly due to SIA or related to associated anomalies or postsurgical complications. Some programs did not collect data on terminations of pregnancy (ETOPFA) or stillbirths, which could lead to underestimation of total prevalence in those regions. However, our methodological analysis (Table 2) indicates that most programs had comprehensive follow‐up mechanisms, including linkage to death certificates up to 5 years, ensuring robust mortality data for surviving infants (Table 5).

Continued surveillance is necessary to monitor trends in SIA prevalence and outcomes. The slight increase in South America's prevalence compared to earlier reports may suggest changes in risk factor exposure or improvements in detection. Additionally, the relatively stable prevalence in Europe and North America implies consistent risk and detection rates over time.

One limitation of this study is the variability in data collection among programs. Although we standardized definitions (e.g., gestational age thresholds for stillbirth), some heterogeneity remains in how cases are reported, especially regarding terminations.

## 5 Conclusions

This study provides a comprehensive analysis of SIA prevalence and mortality over a 41‐year period across 17 countries. The overall prevalence of 2.1 per 10,000 births showed significant regional disparities, with the highest rates observed in Iran (11.5 per 10,000 births) and the lowest in Mexico‐Nuevo León (0.5 per 10,000 births). The increasing prevalence in South America compared to previous reports suggests improved detection, enhanced prenatal screening, and heightened clinical awareness.

Mortality within the first year was 4.3%, primarily driven by associated congenital anomalies, particularly trisomy 21 and complex cardiac defects, reinforcing the strong link between duodenal atresia and Down syndrome. However, non‐congenital factors such as sepsis, aspiration, and anastomotic leaks significantly contributed to mortality, particularly in low‐resource settings with limited access to neonatal intensive care and surgical intervention.

Prenatal diagnosis and elective termination for fetal anomalies (ETOPFA) influenced prevalence rates, with higher termination rates in cases with multiple congenital anomalies (4.6%) and syndromic conditions (3.1%). Despite this, survival outcomes have improved in high‐income countries due to advancements in neonatal care, early surgical intervention, and perioperative management.

It is important to note that information regarding the timing of diagnosis (prenatal vs. postnatal) was not uniformly available across the participating programs. Due to this inconsistency in data collection, we were unable to stratify mortality according to diagnostic timing. We recognize that this distinction provides clinically relevant and epidemiologically meaningful insights, particularly for understanding the impact of prenatal diagnosis on delivery planning, timely access to treatment, and reductions in morbidity and mortality. Therefore, we recommend that future multicenter studies prioritize the systematic and harmonized collection of diagnostic timing in order to enable a more comprehensive analysis of outcome.

Future research should prioritize trend analyses, the impact of prenatal screening on survival, and strategies to reduce mortality in resource‐limited settings. Additionally, long‐term morbidity, neurodevelopmental outcomes, and quality of life in survivors require further evaluation to address global disparities in SIA outcomes.

In conclusion, this multinational analysis provides an updated overview of the epidemiology of SIA. We observed an overall prevalence of approximately 2.1 per 10,000 births and a first‐year mortality of around 4%. There are marked regional differences in prevalence and survival, underlining the influence of healthcare systems and possible genetic/environmental factors. Strengthening surgical care for newborns and addressing regional disparities in healthcare access could further improve outcomes for infants with SIA. Future research should focus on time trends in SIA prevalence and evaluate how early diagnosis (including prenatal screening) and advancements in neonatal surgery contribute to improved survival rates.

## Funding

The authors have nothing to report.

## Ethics Statement

Each institution associated with the congenital anomaly surveillance project of the Latin American Collaborative Study of Congenital Malformations (ECLAMC) obtained approval from the respective institutional ethics committee. All procedures performed in studies involving human participants were conducted in accordance with the ethical standards of the institutional and/or national research committee and with the 1964 Helsinki Declaration and its later amendments or comparable ethical standards.

## Consent

In Latin American countries following the ECLAMC methodology (Poletta et al. 2014), written informed consent was obtained from participants and minors' legal guardians. Since 1992, the EUROCAT Northern Netherlands program (EUROCAT NNL) has required consent, although it was not necessary before; since then, most participants have given their consent. In other countries, participants remain in surveillance programs unless they request removal.

## Conflicts of Interest

The authors declare no conflicts of interest.

## Data Availability

The data that support the findings of this study are available on request from the corresponding author. The data are not publicly available due to privacy or ethical restrictions.

## References

[bdr270032-bib-0001] Adams, S. D. , and M. P. Stanton . 2014. “Malrotation and Intestinal Atresias.” Early Human Development 90, no. 12: 921–925. 10.1016/j.earlhumdev.2014.09.017. 25448782

[bdr270032-bib-0002] Best, K. E. , P. W. Tennant , M. C. Addor , et al. 2012. “Epidemiology of Small Intestinal Atresia in Europe: A Register‐Based Study.” Archives of Disease in Childhood. Fetal and Neonatal Edition 98, no. 5: 353–358. (Corrected and republished from *Archives of Disease in Childhood: Fetal and Neonatal Edition*, 97(5), F353–F358). 10.1136/archdischild-2011-300631. 22933095

[bdr270032-bib-0003] Bethell, G. S. , A. M. Long , M. Knight , and N. J. Hall . 2019. “Congenital Duodenal Obstruction in the UK: A Population‐Based Study.” Archives of Disease in Childhood. Fetal and Neonatal Edition 105, no. 2: 178–183. 10.1136/archdischild-2019-317085. 31229958 PMC7063389

[bdr270032-bib-0004] Bishop, J. C. , B. McCormick , C. T. Johnson , et al. 2020. “The Double Bubble Sign: Duodenal Atresia and Associated Genetic Etiologies.” Fetal Diagnosis and Therapy 47, no. 2: 98–103. 10.1159/000500471. 31167209 PMC6893095

[bdr270032-bib-0005] Burjonrappa, S. , E. Crete , and S. Bouchard . 2011. “Comparative Outcomes in Intestinal Atresia: A Clinical Outcome and Pathophysiology Analysis.” Pediatric Surgery International 27, no. 4: 437–442. 10.1007/s00383-010-2729-8. 20820789

[bdr270032-bib-0006] Cairo, S. , N. Kakembo , P. Kisa , et al. 2017. “Disparity in Access and Outcomes for Emergency Neonatal Surgery: Intestinal Atresia in Kampala Uganda.” Pediatric Surgery International 33, no. 8: 907–915. 10.1007/s00383-017-4120-5. 28677072

[bdr270032-bib-0007] Chirdan, L. B. , A. F. Uba , and S. D. Pam . 2004. “Intestinal Atresia: Management Problems in a Developing Country.” Pediatric Surgery International 20: 834–837. 10.1007/s00383-004-1152-4. 15138787

[bdr270032-bib-0009] England Public Health . 2019. “National Congenital Anomaly and Rare Disease Registration Service (NCARDRS): Congenital Anomalies Statistics 2019: Tables.” https://www.gov.uk/government/publications/ncardrs‐congenital‐anomaly‐annual‐data.

[bdr270032-bib-0010] Estudio Colaborativo Latino Americano de Malformaciones Congénitas (ECLAMC) . n.d. History. http://www.eclamc.org/historia.html. 10.1016/j.rchipe.2016.06.003 27476074

[bdr270032-bib-0011] European Platform on Rare Disease Registration (EUROCAT) . 2019. “Prevalence Charts and Tables—Prevalence Export.” https://eu‐rd‐platform.jrc.ec.europa.eu/eurocat/eurocat‐data/prevalence/export_en.

[bdr270032-bib-0012] Farrokhkhani, P. , R. Farhadi , S. Ala , and S. A. Mousavi . 2023. “Etiology and Outcome of Intestinal Obstruction in Neonates: A 5‐Year Investigation of Admitted Cases From a Tertiary Neonatal Intensive Care Unit in Northern Iran.” Clinical Medicine Insights: Pediatrics 17: 1–5. 10.1177/11795565231196771. PMC10498696 37712054

[bdr270032-bib-0013] Forrester, M. B. , and R. D. Merz . 2004. “Population‐Based Study of Small Intestinal Atresia and Stenosis, Hawaii, 1986–2000.” Public Health 118, no. 6: 434–438. 10.1016/j.puhe.2003.12.017. 15313597

[bdr270032-bib-0014] Galani, A. , A. Zikopoulos , L. Papandreou , E. Mastora , K. Zikopoulos , and G. Makrydimas . 2021. “Prenatal Diagnosis of Fetal Jejunal Atresia: A Case Report.” Cureus 13, no. 10: e18947. 10.7759/cureus.18947. 34815896 PMC8605830

[bdr270032-bib-0015] Garne, E. , L. Rasmussen , and S. Husby . 2002. “Gastrointestinal Malformations in Funen County, Denmark: Epidemiology, Associated Malformations, Surgery and Mortality.” European Journal of Pediatric Surgery 12, no. 2: 101–106. 10.1055/s-2002-30158. 12015653

[bdr270032-bib-0016] Glinianaia, S. V. , J. Rankin , A. Pierini , et al. 2022. “Ten‐Year Survival of Children With Congenital Anomalies: A European Cohort Study.” Pediatrics 149, no. 3: 1–13. 10.1542/peds.2021-053793. 35146505

[bdr270032-bib-0017] Global PaedSurg Research Collaboration . 2021. “Mortality From Gastrointestinal Congenital Anomalies at 264 Hospitals in 74 Low‐Income, Middle‐Income, and High‐Income Countries: A Multicentre, International, Prospective Cohort Study.” Lancet 398, no. 10297: 325–339. 10.1016/S0140-6736(21)00767-4. 34270932 PMC8314066

[bdr270032-bib-0018] Gupta, S. , R. Gupta , S. Ghosh , et al. 2016. “Intestinal Atresia: Experience at a Busy Center of North‐West India.” Journal of Neonatal Surgery 5, no. 4: 51. 10.21699/jns.v5i4.405. 27896159 PMC5117274

[bdr270032-bib-0019] Gutiérrez‐Carrillo, P. M. , J. M. Zertuche‐Coindreau , C. L. Santana‐Cárdenas , C. Esparza‐Ponce , Y. B. Sánchez‐Rodríguez , and J. C. Barrera‐de León . 2013. “Description of the Morbidity and Mortality of Intestinal Atresia in the Neonatal Period.” Cirugia y Cirujanos 81, no. 6: 490–495. https://www.medigraphic.com/cgi‐bin/new/resumen.cgi?IDARTICULO=46773.

[bdr270032-bib-0020] Han, Y. , S. Hu , B. Chen , S. Huang , Q. Quin , and J. Tou . 2022. “Meconium Peritonitis, Intestinal Atresia Combined With Biliary Atresia: A Case Report.” Frontiers in Pediatrics 10: 917116. 10.3389/fped.2022.917116. 35722473 PMC9201381

[bdr270032-bib-0021] Hemming, V. , and J. Rankin . 2007. “Small Intestinal Atresia in a Defined Population: Occurrence, Prenatal Diagnosis and Survival.” Prenatal Diagnosis 27, no. 13: 1205–1211. 10.1002/pd.1886. 17994616

[bdr270032-bib-0022] International Clearinghouse for Birth Defects Surveillance and Research (ICBDSR) . n.d. “International Clearinghouse for Birth Defects Surveillance and Research.” http://www.icbdsr.org/.

[bdr270032-bib-0023] Jiang, J. H. , Y. W. Tsai , S. Y. Lee , and J. H. Chuang . 2022. “Biliary Atresia Associated With Small‐Intestine Atresia: A Condition of High Morbidity and Mortality.” Asian Journal of Surgery 45, no. 10: 1897. 10.1016/j.asjsur.2022.03.130. 35477652

[bdr270032-bib-0024] Khaleghnejad‐Tabari, A. , S. Dastgiri , H. Soori , et al. 2024. “Prevalence of Congenital Anomalies in Iran.” Archives of Iranian Medicine 27, no. 10: 545–550. 10.34172/aim.31287. 39492561 PMC11532651

[bdr270032-bib-0025] Kulkarni, M. 2010. “Duodenal and Small Intestinal Atresia.” Surgery 28, no. 1: 33–37. 10.1016/j.mpsur.2009.10.004.

[bdr270032-bib-0026] Lupo, P. J. , J. L. Isenburg , J. L. Salemi , et al. 2017. “Population‐Based Birth Defects Data in the United States, 2010–2014: A Focus on Gastrointestinal Defects.” Birth Defects Research 109, no. 18: 1504–1514. 10.1002/bdr2.1145. 29152924 PMC5915361

[bdr270032-bib-0027] Martínez‐Frías, M. L. , E. E. Castilla , E. Bermejo , L. Prieto , and I. M. Orioli . 2000. “Isolated Small Intestinal Atresias in Latin America and Spain: Epidemiological Analysis.” American Journal of Medical Genetics 93: 355–359. 10.1002/1096-8628(20000828)93:5<355::aid-ajmg3>3.0.co;2-q. 10951457

[bdr270032-bib-0028] Miscia, M. E. , G. Lauriti , P. Lelli Chiesa , and A. Zani . 2018. “Duodenal Atresia and Associated Intestinal Atresia: A Cohort Study and Review of the Literature.” Pediatric Surgery International 35: 151–157. 10.1007/s00383-018-4387-1. 30386906

[bdr270032-bib-0029] Nanjunda, D. C. , S. J. Lakshmi , H. R. Acharya , and A. K. Mishra . 2022. “Trends in Selected Birth Defects Among Parents From Below Poverty Line Population in Karnataka During 2010–2020.” Indian Journal of Public Health 66, no. 4: 490–493. 10.4103/ijph.ijph_90_22. 37039179

[bdr270032-bib-0030] Nawaz, A. , H. Matta , M. Hamchou , A. Jacobez , O. Trad , and A. H. Al Salem . 2005. “Situs Inversus Abdominus in Association With Congenital Duodenal Obstruction: A Report of Two Cases and Review of the Literature.” Pediatric Surgery International 21, no. 7: 589–592. 10.1007/s00383-005-1412-y. 16012841

[bdr270032-bib-0031] Oddsberg, J. , Y. Lu , and J. Lagergren . 2012. “Aspects of Esophageal Atresia in a Population‐Based Setting: Incidence, Mortality, and Cancer Risk.” Pediatric Surgery International 28, no. 3: 249–257. 10.1007/s00383-011-3014-1. 22020495

[bdr270032-bib-0032] Poletta, F. A. , J. A. Gili , and E. E. Castilla . 2014. “Latin American Collaborative Study of Congenital Malformations (ECLAMC): A Model for Health Collaborative Studies.” Public Health Genomics 17, no. 2: 61–67. 10.1159/000356568. 24457546

[bdr270032-bib-0033] Prasad, T. R. , and M. Bajpai . 2000. “Intestinal Atresia.” Indian Journal of Pediatrics 67, no. 9: 671–678. 10.1007/BF02762182. 11028122

[bdr270032-bib-0034] Rich, B. S. , E. Bornstein , and S. E. Dolgin . 2022. “Intestinal Atresias.” Pediatrics in Review 43, no. 5: 266–274. 10.1542/pir.2021-005177. 35490204

[bdr270032-bib-0035] Shaoying, L. , O. M. Faye‐Peterson , and S. Reilly . 2008. “Intestinal Obstruction in a Term Stillborn Infant.” Laboratory Medicine 39, no. 7: 410–412. 10.1309/DPGEAQRAY9D3UVU0.

[bdr270032-bib-0036] Sigmon, D. F. , B. J. Eovaldi , and H. L. Cohen . 2023. “Duodenal Atresia and Stenosis.” StatPearls. https://www.ncbi.nlm.nih.gov/books/NBK470548/. 29261981

[bdr270032-bib-0037] Takahashi, D. , T. Hiroma , S. Takamizawa , and T. Nakamura . 2014. “Population‐Based Study of Esophageal and Small Intestinal Atresia/Stenosis.” Pediatrics International 56, no. 6: 838–844. 10.1111/ped.12359. 24730728

